# Effects of a Natural Polyherbal Extract on Alleviating Scopolamine-Induced Memory Deficits in C57BL/6 Mice via Enhancing Cholinergic Function

**DOI:** 10.3390/cimb47100817

**Published:** 2025-10-02

**Authors:** Hyeokjin Kwon, Min Ho Kwon, Myeongguk Jeong, Yeeun Kim, Hae-Gyung Yoon, Yeongdon Ju, Kyung-Yae Hyun, Go-Eun Choi

**Affiliations:** 1 Department of Clinical Laboratory Science, College of Health Sciences, Catholic University of Pusan, Busan 46252, Republic of Korea; ghy8627@gmail.com (H.K.); audrnr04@gmail.com (M.J.); 2 Next-Generation Industrial Field-Based Specialist Program for Molecular Diagnostics, Brain Busan 21 Plus Project, Graduate School, Catholic University of Pusan, Busan 46252, Republic of Korea; 3 Basic Medical College, Shaanxi University of Chinese Medicine, Xianyang, Shaanxi 712046, China; ick.minho@gmail.com; 4 Miion Bio Co., Ltd., Seoul 08591, Republic of Korea; 5 Department of Translational Biomedical Sciences, Graduate School of Dong-A University, Busan 49201, Republic of Korea; yeeun0509@naver.com; 6 Division of General Education, Dong-Eui University, Busan 46252, Republic of Korea; hgyoon@deu.ac.kr; 7 Department of Biomedical Laboratory Science, Gimcheon University, Gimcheon 39528, Republic of Korea; 8 Department of Clinical Laboratory Science, Dong-Eui University, Busan 46252, Republic of Korea

**Keywords:** scopolamine, tacrine, Alzheimer’s disease, acetylcholine, natural polyherbal extract

## Abstract

Alzheimer’s disease (AD) is a progressive neurological condition with limited effective pharmaceutical treatments, often accompanied by side effects. This has increased interest in plant-based alternatives. This study examined the cognitive effects of a Natural Polyherbal Extract (NPX) on scopolamine-induced memory deficits in mice. Male C57BL/6 mice (10 weeks old, *n* = 36) were divided into four groups: control (saline), scopolamine (1 mg/kg, i.p.), tacrine (10 mg/kg, oral), and NPX (1000 mg/kg, oral). NPX and tacrine were administered daily by oral gavage for two weeks. Cognitive function was assessed weekly using the Y-maze task. Brain tissues were collected for biochemical analysis, including AChE activity and immunohistochemical detection of neurodegeneration-related markers. Results: Mice treated with NPX demonstrated improved spontaneous alternation behavior compared to the scopolamine group. NPX also significantly reduced acetylcholinesterase activity. Immunohistochemistry revealed decreased expression of amyloid-beta (Aβ) and caspase-3, with enhanced choline acetyltransferase levels. These outcomes were comparable to those observed in the tacrine-treated group. Conclusions: NPX alleviated scopolamine-induced memory impairment through enhancement of cholinergic signaling and mitigation of neurodegenerative markers. The findings suggest that NPX may serve as a promising plant-derived candidate for managing memory-related disorders, including AD.

## 1. Introduction

Among the types of dementia, Alzheimer’s disease (AD) is the most commonly diagnosed and widespread condition. Defined by its progressive neurodegenerative nature, AD affects people globally. The 2025 AD Facts and Figures is a statistical report that provides data from the United States, and it indicates that AD is the most common cause of dementia in the country. Problems with concentration, memory, language and problem-solving abilities, as well as difficulties understanding thoughts and expressing emotions, are all symptoms of AD [1].

Currently, disease is believed to be caused by the accumulation of beta-amyloid (Aβ), an abnormal protein, outside the neurons, followed by the aggregation of tau protein inside the neurons, causing synaptic damage, which leads to neurological dysfunction and brain cell death [2]. The oldest theory of the causes of AD is the cholinergic hypothesis, and it is also very important in explaining how AD occurs. Acetylcholine (ACh) is an important neurotransmitter involved in memory and learning, among other functions [3,4]. ACh levels can be influenced by the central cholinergic nervous system, which regulates the synthesis and secretion of ACh. Previous studies have shown that ACh is produced by a chemical reaction. This reaction is catalyzed by choline acetyl transferase (ChaT) and uses choline and acetyl-CoA as its substrates, and it is involved in cognitive functions such as attention, thinking ability, learning, and memory [5,6]. Scopolamine, which is used in many studies, is a psychotropic drug used as a standard drug for inducing amnesia [7,8]. Scopolamine acts on muscarinic acetylcholine receptors to interfere with cholinergic nerve signal transmission. In other words, it is a non-specific antagonist that acts on muscarinic acetylcholine receptors [9]. This interference with cholinergic nerve signal transmission causes memory impairment. Due to this mechanism, scopolamine is primarily used to create mouse models that induce cognitive impairment [10]. These models are used to evaluate the cognitive and memory-enhancing effects of new treatments or extracts for AD [11,12]. Spontaneous alternation is a widely used method of measuring exploratory behavior in rodents. Generally, T-shaped or Y-shaped maze experiments are conducted. In this study, spatial memory ability was evaluated using a Y-maze experiment [13,14,15].

The induction of oxidative stress in brain neurons is another characteristic of AD. Scopolamine administration in mice results in cognitive impairment accompanied by changes in the brain’s oxidative stress status [16].

The free radical theory of aging helps to explain the molecular mechanisms underlying the aging process and the pathogenesis of age-related diseases, such as cardiovascular disease, dementia, and diabetes. Ascorbic acid, a primary antioxidant, has various beneficial effects on the redox oxidation pathway of the immune system, the mitochondrial pathway, inflammatory aging, and lipoprotein metabolism; additionally, scopolamine can damage the antioxidant defense mechanisms of cells [17,18,19,20,21,22,23].

Tacrine was the first FDA-approved medication, but it has serious limitations for long-term use due to toxicity issues such as liver toxicity, emphasizing the ongoing need for alternative treatments [24,25]. Tacrine is a reversible acetylcholinesterase (AChE) inhibitor that acts on the central nervous system. It is one of the few treatments with proven clinical efficacy in the treatment of AD through AChE inhibition [26]. Tacrine not only inhibits AChE but also increases the synthesis and release of ACh and regulates muscarinic receptors [27]. Tacrine was discontinued in 2013 due to its hepatotoxicity. However, research on new tacrine derivatives without hepatotoxicity is steadily progressing [28].

The progressive loss of cognitive and physical function that characterizes AD is a neurodegenerative disease. It is a chronic condition that can result in significant functional impairment, emotional distress, and a profound impact on the quality of life for both patients and their families. Consequently, the development of effective AD treatments is a pressing task that demands the collective attention of the scientific community. In this study, we evaluated the neuroprotective and antioxidant effects of a natural polyherbal extract (NPX), administered orally to C57BL/6 mice with memory impairment caused by scopolamine. NPX is composed of broccoli powder, kohlrabi powder, radish powder, Gastrodia elata, cultivated wild ginseng root, and concentrated broccoli extract.

## 2. Materials and Methods

### 2.1. Experimental Setup and Treatment Schedule in C57BL/6 Mice

**Figure 1:**
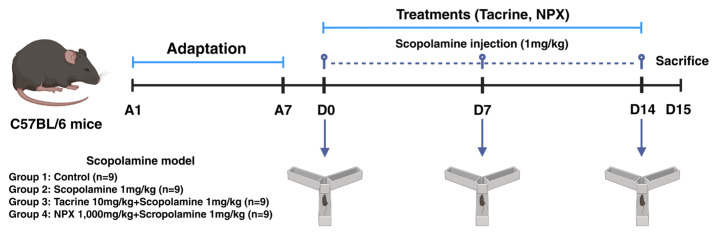
Intraperitoneal and oral administration schedule and behavioral type experiment plan. The figure created with BioRender.com.

For this study, C57BL/6 mice were acclimated to the experimental environment for 1 week before the experiment commenced. The Y-maze test was performed on days 0, 7, and 14 (D0, 7, 14). Scopolamine was administered intraperitoneally 30 min before the test, and tacrine and NPX were administered orally daily (D0–14) (Figure 1).

Approval for all experimental procedures was granted by the Institutional Animal Care and Use Committee of the Catholic University of Pusan (approval code: CUP AEC 2024-003; approval date: 21 October 2024).

### 2.2. Components of NPX

The NPX used in this study is Cell neuron, supplied by Mion Bio Co., Ltd., and its Product registration number is 20030607215231. The composition includes 26.5% broccoli powder, 26.6% kohlrabi powder, 26.5% radish powder, 13% Gastrodia elata, 6.7% cultured wild ginseng root, and 0.6% broccoli concentrate. NPX has received international food safety certification from the Food Safety System Certification Foundation (FSSC22000, Netherlands) and FFR (Food Facility Registration, U.S., FDA) certification.

### 2.3. Animals and Treatments

We used 36 male C57BL/6 mice, 10 weeks old, supplied by Kosa Bio (Sungnam, Republic of Korea). Mice weighing 26 ± 2 g at the beginning of the experiment were used. Specific pathogen-free (SPF) mice were housed in a ventilated laboratory. The temperature was maintained at 22 ± 2 °C, humidity at 50 ± 10%, and a 12 h light/dark cycle was applied. All mice were provided with standard feed and water ad libitum. All experimental procedures were conducted after obtaining approval from the Institutional Animal Care and Use Committee of the Catholic University of Pusan (CUP AEC 2024-003). All mice underwent a 1-week adaptation period, after which a randomized complete block design was employed to allocate them into four experimental groups (Table 1).

The experimental setup involved several distinct groups. Group 1 (Con) received only an equal volume of saline orally, ensuring they did not experience cognitive dysfunction. To induce cognitive impairment, the remaining three groups were administered 1 mg/kg of scopolamine (S0929, Sigma-Aldrich Co., St. Louis, MO, USA) intraperitoneally 30 min before each behavioral test. Among these scopolamine-treated groups, group 3 (PC) received 10 mg/kg of tacrine (A79922, Sigma-Aldrich, St. Louis, MO, USA) orally, while group 4 (NPX) received 1000 mg/kg of NPX orally. These test substances (tacrine and NPX), prepared in DPBS, were administered orally once daily for two weeks at a consistent time. This daily oral regimen was intentionally kept distinct from the behavioral tests, specifically to avoid any acute pharmacological effects, allowing us to assess the influence of NPX on cognitive capabilities after two weeks of treatment.

### 2.4. Y-Maze Test

In this study, scopolamine was administered intraperitoneally 30 min prior to each Y-maze assessment. The experimental apparatus comprised a Y-shaped structure featuring three white plastic arms. Each arm extended 43 cm in length, was 16 cm high, and 10 cm wide, with a 120° angle separating each of the three arms. These arms were designated A, B, and C. For testing, individual mice were positioned at the entrance of one arm, and their locomotor activities were documented. The sequence and frequency of entries into each arm were meticulously logged for every mouse during a 1 min observation period. Percent alternation was defined as the number of spontaneous alternations divided by the total possible triads. Spontaneous alternation was calculated by applying the formula shown in Equation (1) below. For example, in this maze, if the mice enter the arms in the following order: B, C, A, C, A, B, it was scored as having two spontaneous alternations (BCA and CAB) out of four possible triads (BCA, CAC, ACA, and CAB), resulting in 50% alternation. Standard conditions for evaluating learning and memory abilities were established, and their rationality, accuracy, and reproducibility were verified by considering the test methodology.

(1) $$\mathrm{Spontaneous}\;\mathrm{alternation}\;(\%)=\frac{actual\;alteration}{maximum\;alternation}\times 100$$

### 2.5. Serum Measurement Methods

Once the experiment concluded, CO_2_ gas was administered for mouse euthanasia. Blood was subsequently collected via cardiac puncture, and serum was isolated by centrifuging the samples at 3000 rpm for 10 min at 4 °C.

#### 2.5.1. Serum Ascorbic Acid Concentration Analysis

Antioxidant activity was analyzed using the EZ-Ascorbic Acid Assay Kit (DG-ASC100, DoGenBio, Seoul, Republic of Korea). A 100 μL sample of the prepared serum was dispensed onto a 96-well plate and reacted at room temperature (RT) for 15 min. Then, the reaction mix (100 μL) was added to each well. The wells contained the sample or standard solution. Absorbance readings were acquired at 593 nm within 2–3 min with a Varioskan LUX multimode microplate reader (Thermo Fisher Scientific, Waltham, MA, USA). We calculated antioxidant capacity using the ascorbic acid standard curve that the colorimetric kit provided.

#### 2.5.2. Serum AChE Activity Analysis

The inhibitory effect on AChE activity was assessed utilizing an EZ-Acetylcholinesterase Assay Kit (DG-ACE100, DoGenBio, Seoul, Republic of Korea). For this, 5 µL of mouse serum was aliquoted into a 96-well plate, and the final volume was brought to 50 µL using assay buffer. Following this, 50 µL of the reaction mixture was dispensed into each well and incubated in the dark at RT for a duration of 30 min. Absorbance readings were subsequently acquired at 570 nm with a Varioskan LUX multimode microplate reader (Thermo Fisher Scientific, Waltham, MA, USA). AChE activity was determined based on the standard curve provided by the colorimetric kit. We calculated AChE activity using the standard solution curve that the colorimetric kit provided.

### 2.6. Immunohistochemistry Staining Method

Choline acetyltransferase (ChaT), beta-amyloid, and caspase-3 expression levels were evaluated using IHC. After 24 h of fixation in paraformaldehyde, the brain tissues underwent a graded dehydration process using alcohol solutions prior to paraffin embedding. Subsequently, 4 μm thick sections were prepared from the brain tissue, mounted onto slides, air-dried, and then cured in an oven at 65 °C for 2 h. The antibodies used in this study are as follows. Primary antibodies utilized included anti-ChaT (SC-55557), anti-Aβ (SC-28365), and anti-caspase-3 (SC-7272), all sourced from Santa Cruz (Dallas, TX, USA) and applied at a 1:200 dilution. Incubation with these primary antibodies occurred overnight at 4 °C. Subsequently, a 1:200 dilution of Goat anti-rabbit IgG H&L (ab150077, Abcam Ltd., Cambridge, UK) was applied, followed by a 30 min incubation at RT. Immunoreactivity was visualized using the DAB Substrate Kit (SK-4100, Vector Laboratories, Burlingame, CA, USA), in accordance with the manufacturer’s guidelines. Subsequently, the tissue sections underwent extensive washing under running tap water, followed by counterstaining with hematoxylin for a duration of 1 to 5 min.

Quantitative morphological analysis was conducted utilizing ImageJ v1.53 (National Institutes of Health, Bethesda, MD, USA). The generated results were subsequently illustrated in the format of a bar graph. Histological assessment was performed on three separate tissue sections.

### 2.7. Statistical Analysis

The data presented herein are the result of a minimum of three independent experiments and are expressed as the mean ± standard deviation (SD). Statistical analyses were conducted using GraphPad Prism 8 software (version 8.4.3, GraphPad Software Inc., San Diego, CA, USA). One-way analysis of variance (ANOVA) was applied to evaluate differences among groups. For subsequent multiple comparisons, post hoc Bonferroni tests were performed. Statistical significance was set at a *p*-value threshold of less than 0.05.

## 3. Results

### 3.1. Improving Cognitive Function by Increasing Y-Maze Scores

**Figure 2:**
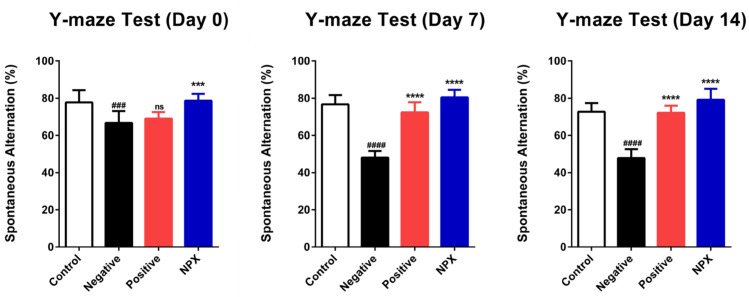
Effects of NPX on Spatial Cognition (Y-Maze Test). This figure presents data from the Y-maze test conducted on Day 0, Day 7, and Day 14. Data are expressed as mean ± S.D. (*n* = 9). *** *p* < 0.001, **** *p* < 0.0001 compared with the NC group. ^###^ *p* < 0.001, ^####^ *p* < 0.0001 compared with the control group. ns indicates no statistically significant difference (*p* ≥ 0.05).

The Y-maze test results indicated the successful induction of cognitive impairment. On Day 0, the NC group scored significantly lower compared to the control group (*p* < 0.001). While the PC group showed no significant difference from the NC group on Day 0 (ns, *p* ≥ 0.05), it exhibited a significant increase in performance on Days 7 and 14 when compared to the NC group (*p* < 0.0001). The NPX group consistently demonstrated a significant increase in performance over the NC group across all assessment days (Days 0–14, *p* < 0.0001). These findings collectively suggest that NPX may help restore impaired spatial cognition (Figure 2).

### 3.2. Measuring Serum

The effects of NPX on serum antioxidant capacity and AChE activity were evaluated in mice showing memory dysfunction induced by scopolamine.

#### 3.2.1. Ascorbic Acid Concentration

**Figure 3:**
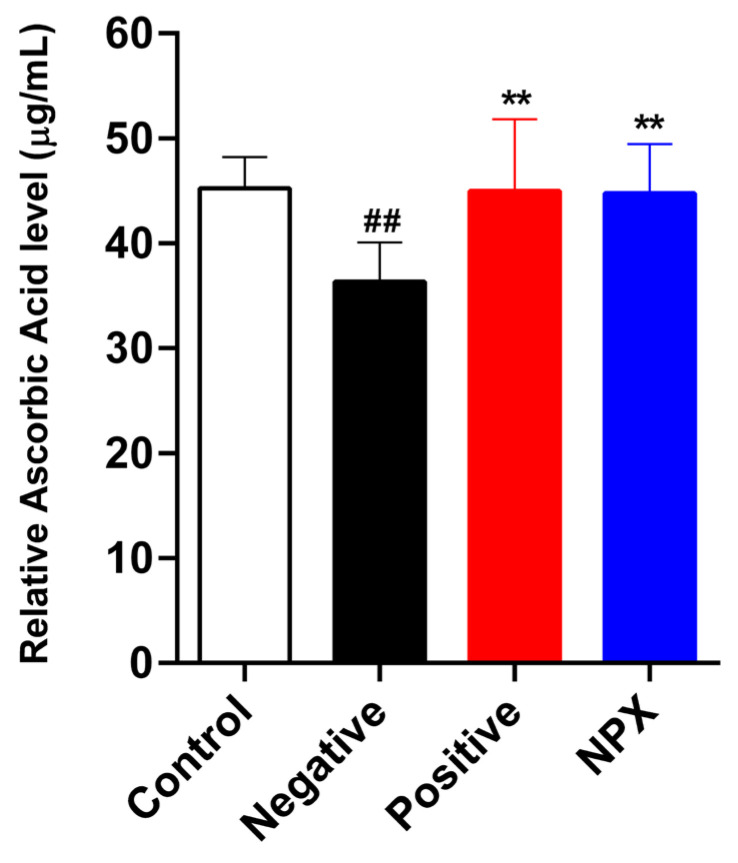
Effects of NPX on Serum Ascorbic Acid Concentration. This figure presents the serum ascorbic acid concentration across different experimental groups. Data are expressed as mean ± S.D. (*n* = 9). ** *p* < 0.01 compared with the NC group. ^##^ *p* < 0.01 compared with the control group.

The analysis of serum ascorbic acid concentration revealed that the scopolamine-treated NC group exhibited a significantly lower concentration compared to the control group (*p* < 0.01). This finding suggests a depletion of antioxidant reserves within the scopolamine-induced memory impairment model. Conversely, a substantial elevation in serum ascorbic acid levels was observed in both the PC group (*p* < 0.01) and the NPX group (*p* < 0.01) when compared to the NC group. These observations collectively indicate that NPX effectively mitigates the scopolamine-induced decline in ascorbic acid, demonstrating an efficacy comparable to that of tacrine (Figure 3).

#### 3.2.2. Acetylcholinesterase Activity

**Figure 4:**
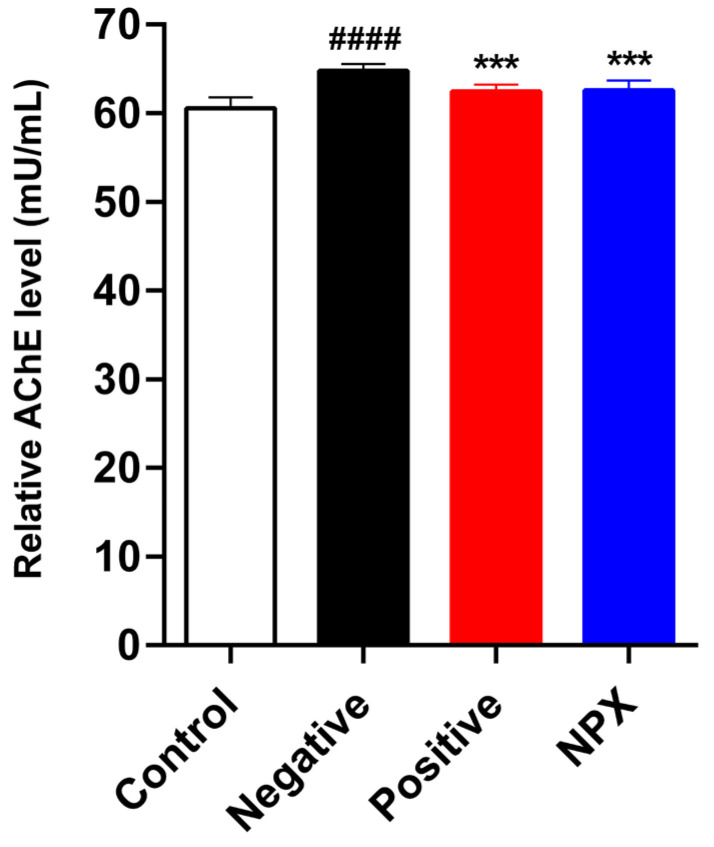
Effects of NPX on AChE Activity. This figure presents the AChE activity across different experimental groups. Data are expressed as mean ± S.D. (*n* = 9). *** *p* < 0.001 compared with the NC group. ^####^ *p* < 0.0001 compared with the control group.

The experimental results demonstrated a significant elevation in AChE activity within the NC group (*p* < 0.0001) when compared to the control group. This finding aligns with prior research indicating that scopolamine accelerates cognitive decline through the promotion of AChE activity and excessive acetylcholine degradation in the synaptic cleft. Furthermore, the AChE activity observed in the PC group (*p* < 0.001) provided clear confirmation of tacrine’s pharmacological efficacy as an AChE inhibitor. Notably, the NPX group (*p* < 0.001) also exhibited a significant reduction in AChE activity, mirroring the effect seen in the PC group. These findings collectively suggest that NPX possesses AChE-inhibiting properties comparable to those of tacrine (Figure 4).

### 3.3. Immunohistochemistry Staining

#### 3.3.1. Choline Acetyltransferase (ChaT)

**Figure 5:**
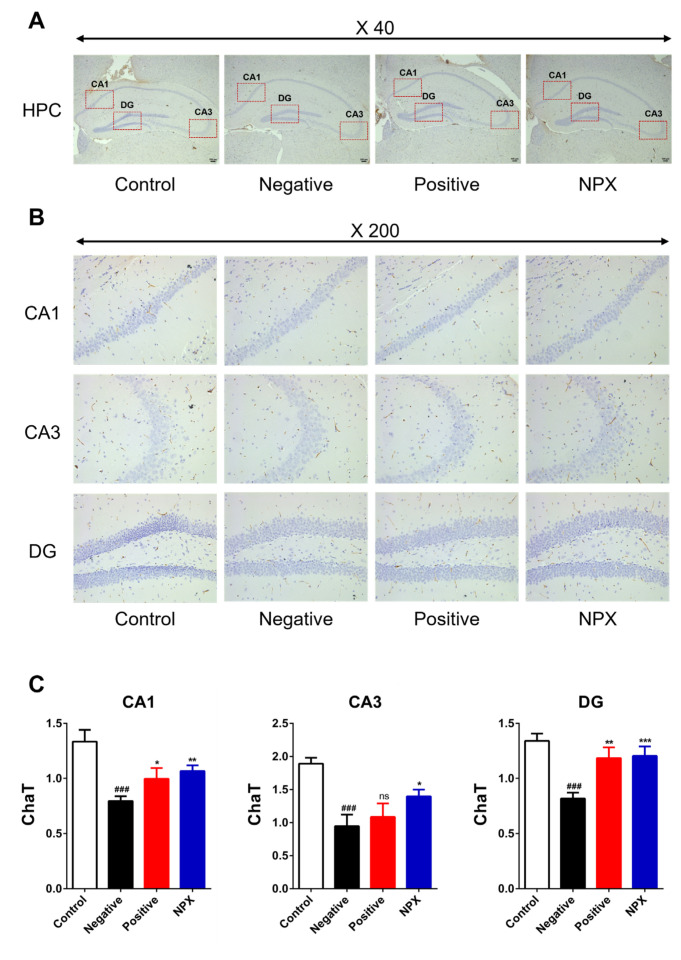
Effects of NPX on ChaT Protein Expression in Hippocampal Regions. This figure presents ChaT protein expression levels in hippocampal subregions (CA1, CA3, DG) across different experimental groups. (**A**) Representative photomicrograph of the hippocampus at ×40 magnification. (**B**) Representative photomicrographs of CA1, CA3, and DG subregions at ×200 magnification. (**C**) Quantification of ChaT protein expression in CA1, CA3, and DG. Data are expressed as mean ± S.D. (*n* = 9). * *p* < 0.05, ** *p* < 0.01, *** *p* < 0.001 compared with the NC group; ^###^ *p* < 0.001 compared with the control group. ns indicates no statistically significant difference (*p* ≥ 0.05).

ChaT, critical for ACh synthesis and efficient cholinergic neurotransmission, was significantly reduced in its expression within the NC group (*p* < 0.001) compared to the control group. This reduction suggests impaired cholinergic function. In contrast, the NPX group exhibited a significant restoration of ChaT expression across all hippocampal regions (CA1: *p* < 0.01, CA3: *p* < 0.05, DG: *p* < 0.001), indicating its potential to enhance cholinergic neurotransmission. The PC group also demonstrated a trend towards recovery in ChaT expression in some regions (CA1: *p* < 0.05, DG: *p* < 0.01), though no statistically significant recovery was observed in the CA3 region (ns ≥ 0.05) (Figure 5).

#### 3.3.2. Amyloid β-Peptide (Aβ)

**Figure 6:**
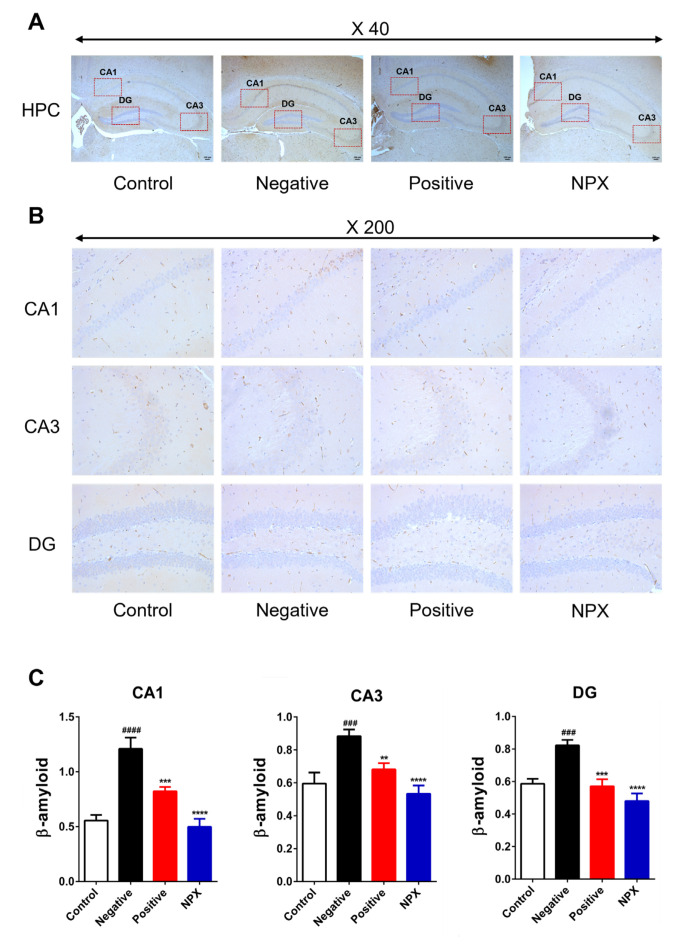
Effects of NPX on Aβ Accumulation in Hippocampal Regions. This figure illustrates Aβ levels in hippocampal subregions (CA1, CA3, DG) across different experimental groups. (**A**) Representative photomicrograph of the hippocampus at ×40 magnification. (**B**) Representative photomicrographs of CA1, CA3, and DG subregions at ×200 magnification. (**C**) Quantification of Aβ accumulation in CA1, CA3, and DG. Data are expressed as mean ± S.D. (*n* = 9). ** *p* < 0.01, *** *p* < 0.001, **** *p* < 0.0001 compared with the NC group; ^###^ *p* < 0.001, ^####^ *p* < 0.0001 compared with the control group.

This study assessed the impact of NPX on Aβ accumulation. Aβ levels in the scopolamine-treated NC group showed a significant increase compared to the control group across hippocampal regions (CA1: *p* < 0.0001; CA3, DG: *p* < 0.001). This suggests that Aβ accumulation occurs as a neuropathological consequence of scopolamine administration. Notably, both the PC group (CA1, DG: *p* < 0.001; CA3: *p* < 0.01) and the NPX group (CA1, CA3, DG: *p* < 0.0001) exhibited a significant reduction in Aβ levels within the CA1, CA3, and DG regions of the hippocampus when compared to the NC group (Figure 6).

#### 3.3.3. Caspase-3 (Cas-3)

**Figure 7:**
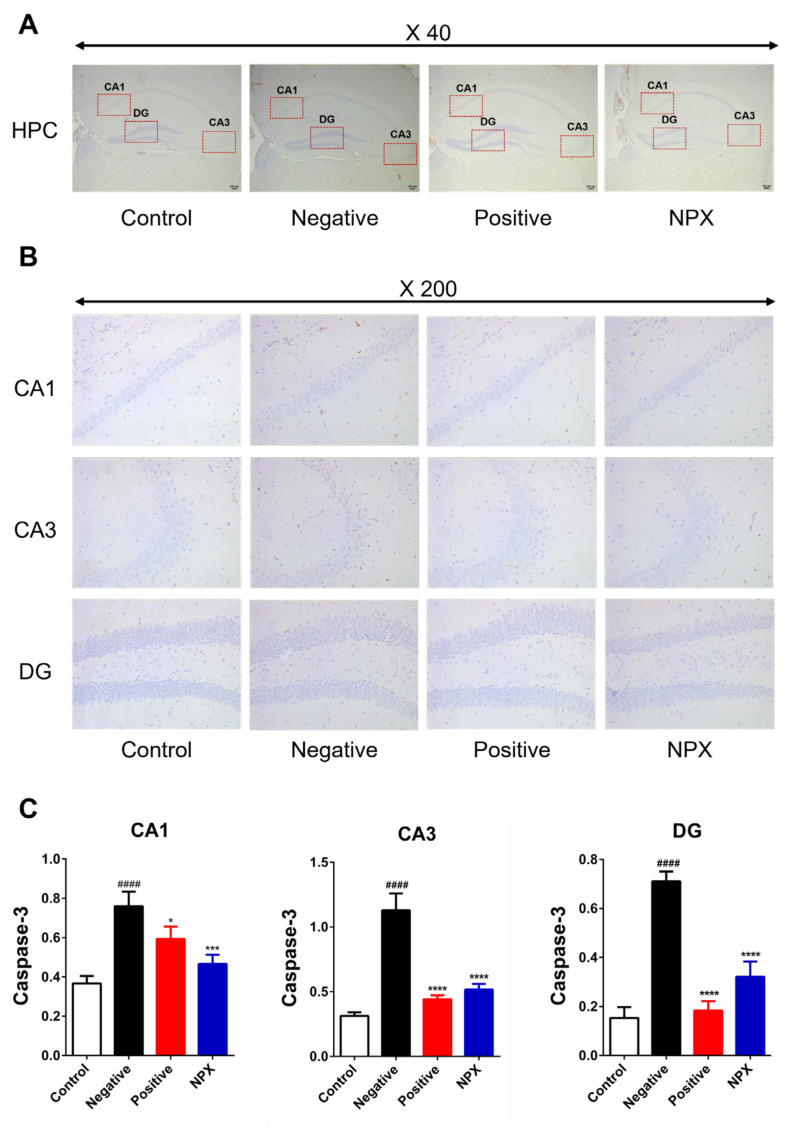
Effects of NPX on Cas-3 Expression in Hippocampal Regions. This figure illustrates Cas-3 expression levels in hippocampal subregions (CA1, CA3, DG) across different experimental groups. (**A**) Representative photomicrograph of the hippocampus at ×40 magnification. (**B**) Representative photomicrographs of CA1, CA3, and DG subregions at ×200 magnification. (**C**) Quantification of Cas-3 expressions in CA1, CA3, and DG. Data are expressed as mean ± S.D. (*n* = 9). * *p* < 0.05, *** *p* < 0.001, **** *p* < 0.0001 compared with the NC group; ^####^ *p* < 0.0001 compared with the control group.

This study assessed the impact of NPX on Cas-3 activity inhibition. As a result, the NC group exhibited a significant increase in Cas-3 expression across all hippocampal regions (CA1, CA3, DG: *p* < 0.0001) compared to the control group. This suggests that scopolamine administration induced apoptosis signaling. Notably, both the PC group (CA1: *p* < 0.05, CA3, DG: *p* < 0.0001) and the NPX group (CA1: *p* < 0.001, CA3, DG: *p* < 0.0001) showed a significant decrease in Cas-3 levels within the CA1, CA3, and DG regions of the hippocampus when compared to the NC group (Figure 7).

## 4. Discussion

This study aimed to investigate the cognitive-enhancing effects and underlying mechanisms of a natural polyherbal extract (NPX) in a mouse model of scopolamine-induced memory impairment. The scopolamine model is commonly used to mimic the pathological features of Alzheimer’s disease (AD), which are characterized by dysfunction of the cholinergic neurotransmitter system and increased oxidative stress [29,30]. We evaluated the effects of NPX on serum antioxidant capacity, cholinergic markers, beta-amyloid accumulation, and apoptotic indicators and compared them with the effects of tacrine. Our experimental results showed that the expression of choline acetyltransferase (a key enzyme in cholinergic neurotransmission) was significantly reduced in the CA1, CA3, and dentate gyrus (DG) regions of the hippocampus in the scopolamine-treated negative control (NC) group [31,32,33]. The results show that scopolamine hampers the production of acetylcholine, thus weakening the transmission of the neurotransmitter acetylcholine. Concurrently, AChE activity increased significantly in the NC group, suggesting accelerated acetylcholine degradation and reduced synaptic acetylcholine levels [34,35]. These disruptions to the cholinergic system align with the primary pathological mechanisms of scopolamine-induced memory impairment.

Furthermore, the concentration of serum ascorbic acid was significantly lower in the NC group, indicating that the oxidative stress induced by scopolamine led to the depletion of endogenous antioxidant substances [36,37,38]. Aβ levels and caspase-3 expression were also significantly higher in the NC group, confirming that scopolamine induced Aβ accumulation and activation of the apoptotic signaling pathway, both of which are major neuropathological hallmarks of AD [35,39]. These findings collectively support that the scopolamine model effectively induces various neuropathological changes akin to those in AD.

Surprisingly, NPX effectively reversed most of the pathological changes induced by scopolamine. The significant increase in serum ascorbic acid concentration and the significant reduction in AChE activity in the NPX group demonstrate that NPX protects cholinergic neurotransmission through its potent antioxidant and AChE inhibitory effects [12,40]. Specifically, the reduction in AChE activity suggests a crucial mechanism by which NPX may contribute directly to cognitive improvement by maintaining or increasing brain acetylcholine levels [41].

Furthermore, immunohistochemical analysis revealed that the NPX group significantly recovered the reduced ChaT expression in all analyzed hippocampal regions (CA1, CA3, and DG). This implies that NPX enhances neurotransmission by promoting acetylcholine synthesis [42]. Furthermore, NPX effectively inhibited the scopolamine-induced increases in Aβ accumulation and Caspase-3 expression, demonstrating neuroprotective effects. The multifaceted effects of NPX were highly comparable to those observed in the tacrine-treated group (PC group). NPX was found to be as effective as tacrine in terms of serum ascorbic acid concentration, AChE activity, Aβ accumulation, and Caspase-3 expression. Notably, NPX significantly increased the recovery of ChaT expression in the CA3 region, whereas tacrine had no significant effect. These results suggest that NPX may be superior to tacrine in certain areas. Taken together, the results of this study strongly suggest that NPX has the potential to improve cognitive function in a mouse model of scopolamine-induced memory impairment. These improvements would be achieved by restoring cholinergic function, reducing oxidative stress, and inhibiting Aβ accumulation.

The multifaceted neuroprotective and cognitive-enhancing effects observed with NPX treatment can be largely attributed to its unique polyherbal composition. NPX is formulated from a combination of broccoli powder, kohlrabi powder, radish powders, *Gastrodia elata*, and cultured wild ginseng root, each known for distinct beneficial properties. For instance, the robust antioxidant activity displayed by NPX, evidenced by increased serum ascorbic acid levels and reduced oxidative markers, can be primarily linked to the rich content of sulforaphane, isothiocyanates, and other diverse antioxidants present in cruciferous vegetables like broccoli, kohlrabi, and radish [43,44,45,46]. Furthermore, NPX’s positive modulation of cholinergic markers (ChaT and AChE activity) and its ability to suppress Aβ accumulation and Caspase-3 activation likely stem from the well-documented neuroprotective, anti-inflammatory, and cognitive-enhancing properties of *Gastrodia elata* and the various ginsenosides found in cultured wild ginseng root [47,48,49,50,51,52,53,54]. This synergistic blend of bioactive compounds in NPX may collectively contribute to its broad spectrum of beneficial effects, effectively targeting multiple pathways implicated in scopolamine-induced memory impairment.

These results suggest that NPX, a naturally occurring substance that modulates the acetylcholine system and neuroprotective mechanisms, could be used to develop new therapeutics for neurodegenerative diseases, such as AD.

## 5. Conclusions

The results of this study demonstrate that NPX exhibits positive effects comparable to those of tacrine in mice with scopolamine-induced memory impairment, a model frequently used to mimic Alzheimer’s disease (AD)-like dementia. Serum tests revealed comparable ascorbic acid concentrations and acetylcholinesterase activity levels to those observed with tacrine treatment. Immunohistochemical (IHC) analysis showed that NPX treatment significantly increased ChaT levels and effectively inhibited beta-amyloid accumulation and caspase-3 activation. These effects are comparable to those observed in the tacrine-treated group. When these results are considered together, NPX appears to improve cognitive function and has effects like those of tacrine, a treatment that was previously used for Alzheimer’s disease. Therefore, NPX shows promise as a new potential treatment for improving cognitive function.

This study has some limitations. It was conducted using a single animal model that primarily reflects cholinergic dysfunction. To address this, future studies should incorporate functional assays using cholinergic neuronal cell models (e.g., SH-SY5Y, or primary hippocampal neurons) and explore the relevant cell signaling pathways to clarify the direct effects of NPX. Such functional assays will provide stronger mechanistic evidence before considering translational studies. Ultimately, well-designed clinical trials will be necessary to establish the efficacy, safety, and clinical potential of NPX in patients with Alzheimer’s disease.

## Figures and Tables

**Table 1:** Experimental Group Design.

Group No.	Designation	Treatment	*n*
Group 1	Con	Saline	9
Group 2	NC	SCP 1 mg/kg	9
Group 3	PC	SCP 1 mg/kg + Tacrine 10 mg/kg	9
Group 4	NPX	SCP 1 mg/kg + NPX 1000 mg/kg	9

## Data Availability

The original contributions presented in this study are included in the article. Further inquiries can be directed to the corresponding authors.

## Abbreviations

The following abbreviations are used in this manuscript:

AD	Alzheimer’s disease
Cas-3	Caspase-3
ChaT	Choline acetyltransferase
AChE	Acetylcholinesterase
ACh	Acetylcholine
HPC	Hippocampus
Acetyl-CoA	Acetyl-coenzyme A
Aβ	Beta-amyloid
NPX	Natural polyherbal extract
NC	Negative control
PC	Positive control
RT	Room temperature
